# Hysteroscopic Resection for Placental Polyp With Temporary Laparoscopic Uterine Artery Clipping

**DOI:** 10.7759/cureus.88689

**Published:** 2025-07-24

**Authors:** Kanato Yoshiike, Takashi Suzuki, Kenta Sonehara, Hiroshi Nabeshima, Osamu Oguchi

**Affiliations:** 1 Obstetrics and Gynaecology, Saku Central Hospital, Saku, JPN

**Keywords:** hysteroscopic surgery, laparoscopic technique, miscarriage, placental polyps, surgical management of obstetrical hemorrhage

## Abstract

Placental polyps, consisting of residual trophoblastic tissue after delivery or miscarriage, are also known as a type of retained products of conception (RPOC). This is a rare but potentially severe condition that can cause significant hemorrhage, which can be life-threatening. Uterine artery embolization (UAE) followed by hysteroscopic transcervical resection (TCR) is used as a uterine-preserving intervention. However, the UAE may not be ideal for patients seeking a future pregnancy because of the potential risks of uterine ischemia and infection. Herein, we report a case of a 38-year-old woman with a placental polyp and hemorrhagic shock that developed after a miscarriage. Following the transfusions of eight units of red blood cells and four units of fresh frozen plasma, the patient recovered. Transvaginal ultrasonography revealed a hypervascular mass in the uterine cavity, suggestive of a placental polyp. We performed temporary laparoscopic uterine artery clipping to minimize bleeding, followed by TCR. This procedure may be an effective alternative to UAE for preventing long-term uterine ischemia and preserving fertility.

## Introduction

Placental polyps are intrauterine masses of residual trophoblastic tissues and the most common form of retained products of conception (RPOC) that develop after delivery or miscarriage. The incidence of placental polyps is less than 0.25% in all pregnancies [1], but they may cause massive hemorrhage requiring blood transfusions, uterine artery embolization (UAE), hysteroscopic resection, and even hysterectomy. The efficacy of UAE followed by hysteroscopic transcervical resection (TCR) as a uterine-preserving treatment [2] has been previously reported. Although the frequency of this combined approach has not been well established in the literature, it is often considered in cases with hypervascular retained products of conception. However, the UAE may not be suitable for patients who desire fertility preservation due to the risk of uterine ischemia and infection. Complete resection of placental polyps by TCR may be difficult to achieve without UAE because the hysteroscopic view is obstructed by hemorrhage. Temporary intraoperative occlusion of the uterine artery during myomectomy has been shown to reduce blood loss and provide favorable outcomes for subsequent pregnancy [3]. We report a case of laparoscopic uterine artery clipping and hysteroscopic resection of a placental polyp.

## Case presentation

A 38-year-old woman (gravida two, para one vaginal delivery) conceived by in vitro fertilization-embryo transfer (IVF-ET) but was diagnosed with miscarriage at eight weeks of gestation. She had a 7 cm fibroid on the anterior wall of the uterus. She was referred to our hospital for treatment of a miscarriage. We suggested evacuation, but she did not undergo any intervention. She was brought to the emergency room by ambulance because of massive bleeding six weeks after the diagnosis of miscarriage, with no history of any medical or surgical intervention at another facility during that time. She appeared pale and peripherally shut down, with a pulse rate of 130 beats/min and blood pressure of 79/60 mmHg. The gestational sac was discharged into the uterine cervical canal, indicating progressive miscarriage, and was removed using placental forceps. Her hemoglobin level was 6.3 g/dL, and fibrinogen level was 181 mg/dL (Table 1); therefore, eight units of red blood cells and four units of fresh frozen plasma were transfused. The vaginal bleeding stopped, and the patient’s condition improved. The patient was discharged three days after admission.

**Table 1:** Blood investigation results WBC: white blood cell, Hb: hemoglobin, PLT: platelet, PT: prothrombin time, APTT: activated partial thromboplastin time, Fib: fibrinogen, Alb: albumin, T-bill: total bilirubin, AST: aspartate aminotransferase, ALT: alanine aminotransferase, LDH: lactate dehydrogenase, BUN: blood urea nitrogen, Cre: creatinine, CRP: C-reactive protein, hCG: human chorionic gonadotropin

Blood Investigation	Result	Normal Range
WBC	23,100	3,300-8,600 /μL
Hb	6.3	11.6-14.8 g/dL
PLT	38.0x10^4^	15.8-34.8x10^4^/μL
PT	13.5	10.0-13.5 sec
APTT	27.6	24.0-34.0 sec
Fib	181	200-400 mg/dL
Alb	2.8	4.1-5.1 g/dL
T-bill	0.5	0.4-1.5 mg/dL
AST	13	13-30 U/L
ALT	9	7-23 U/L
LDH	119	124-222 U/L
BUN	15	8-20 mg/dL
Cre	0.4	0.46-0.79 mg/dL
Na^+^	136	138-145 mEq/L
K^+^	3.3	3.6-4.8 mEq/L
Cl^-^	108	101-108 mEq/L
CRP	0.66	0.00-0.14 mg/dL

**Figure 1:**
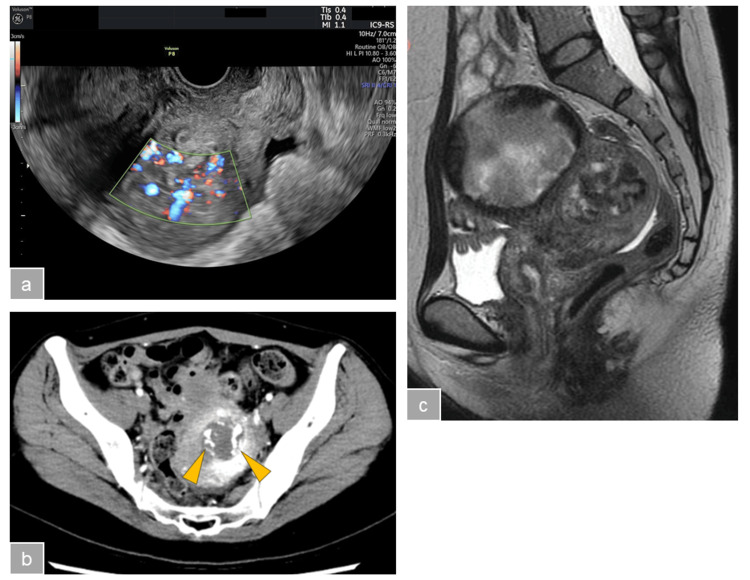
Preoperative imaging A) Transvaginal ultrasonography shows intrauterine mass with prominent blood flow. B) Contrast-enhanced computed tomography (CT) scan shows a hypervascular mass in the uterine cavity that is mainly perfused by the uterine artery (arrowhead). C) T2-weighted magnetic resonance imaging (MRI) scan shows a degenerated myoma in the anterior wall of the uterus, and a 4.5 cm sized heterogeneous mass in the uterine cavity was confirmed.

However, two weeks later, she was readmitted to our hospital because of genital bleeding. Her human chronic gonadotropin (hCG) level was 10.0 mIU/mL, and transvaginal ultrasonography revealed a hypervascular mass in the uterine cavity (Figure 1A). Contrast-enhanced computed tomography (CT) and magnetic resonance imaging (MRI) scanning were performed. An intrauterine mass of 45 × 30 mm, perfused mainly by the uterine artery. No structures suspicious for arteriovenous malformation (AVM) were observed, and a placental polyp was suspected (Figures 1B, 1C).

**Figure 2:**
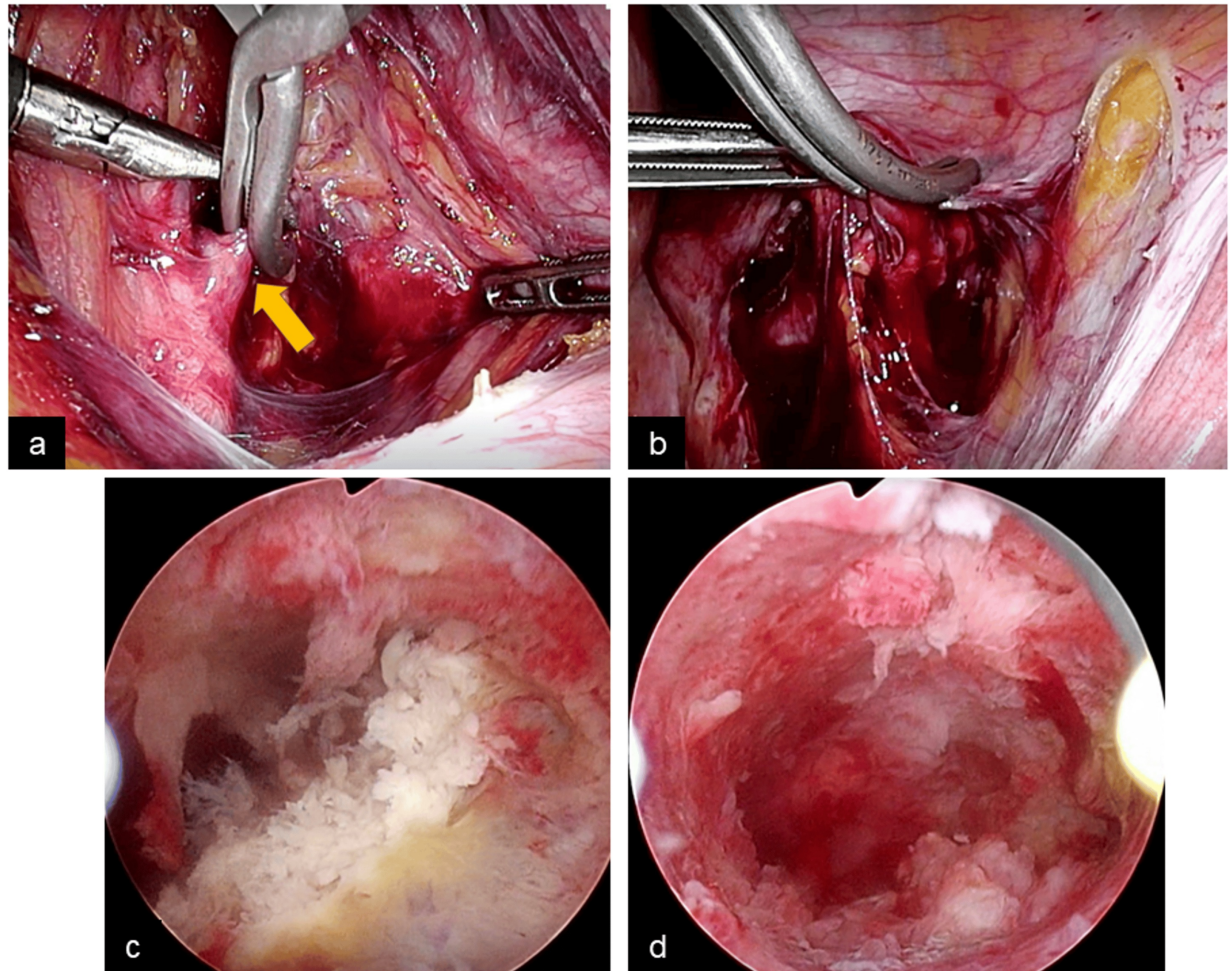
Intraoperative laparoscopic and hysteroscopic imaging A) Laparoscopic view showing left uterine artery clipping (arrow). B) Laparoscopic view showing the right uterine artery clipping. C) Hysteroscopic view before the resection of placental polyp. D) Hysteroscopic view after the resection.

As the patient desired fertility preservation, we decided to perform TCR to remove the placental polyps. Laparoscopic uterine artery clipping was performed under general anesthesia to reduce intraoperative bleeding using a 5 mm trocar in each bilateral lower abdomen and a 12 mm trocar in the umbilicus and median lower abdomen. The broad ligament was incised, and the bilateral uterine arteries were identified and occluded respectively with 10 mm vascular clips (AESCULAP Endo Temporary Clip, B. Braun, Melsungen, Germany). It took 16 and 13 minutes to isolate the left and right uterine arteries, respectively (Figures 2A, 2B).

We then performed TCR using a bipolar resectoscope (OES Pro Resectoscope, OLYMPUS, Tokyo, Japan) and removed the placental polyps (Figure 2C). The clip was removed laparoscopically, and no hemostasis in the uterus was confirmed using hysteroscopy (Figure 2D). The broad ligament was sutured to prevent adhesions. The operative time was 149 min, with minimal bleeding, and the uterine artery occlusion time was 80 min.

Concurrent myomectomy was not performed, considering the risk of uterine perforation or infection, which may cause uterine rupture in the next pregnancy. The postoperative course was uneventful, and the patient was discharged three days postoperatively. Histological examination revealed degenerated chorionic villi and trophoblastic tissues. The patient resumed menstruation 30 days postoperatively.

Written informed consent was obtained from the patient for the publication of this study.

## Discussion

This case report demonstrates the successful application of laparoscopic uterine artery clipping and TCR for placental polyps. Although an increasing number of cases of placental polyps have been reported, their management has not yet been established due to a lack of evidence. Assessment of blood flow by color Doppler has been reported to be useful in predicting the risk of massive hemorrhage [4]. In vitro fertilization and embryo transfer (IVF-ET) pregnancies are associated with the development of placental polyps and hemorrhagic shock [5,6]. Clinicians should be aware that even early miscarriages involve a risk of massive bleeding, especially in IVF-ET pregnancies. The number of placental polyps may increase with assisted reproductive technology (ART) pregnancies. Certain hemostasis and fertility preservation measures are necessary for subsequent pregnancy.

While the UAE has demonstrated efficacy in preserving the uterus, uterine artery clipping is considered an effective alternative, as it avoids radiation exposure, reduces intraoperative blood loss, and prevents long-term uterine ischemia. The technique for identifying and clipping the uterine artery is simple if the surgeon is accustomed to performing laparoscopic surgery. This procedure also has the advantage that uterine perforations can be observed laparoscopically and can be performed in facilities where UAE is not immediately available. Intra-aortic balloon occlusion can also be an effective option for temporarily blocking blood flow. However, it carries risks such as ischemia of the lower limb and dislocation of the inguinal sheath or the placed balloon when changing positions during surgery.

TCR without UAE results in a high frequency of major bleeding in cases with an abundant blood supply, while TCR with UAE is effective for placental polyps with strong vascularity [7]. Takeuchi et al. reported that of 13 patients who underwent TCR after UAE for placental polyps, 11 wished to conceive and eight became pregnant with a good pregnancy outcome [8]. Although UAE is a uterine-preserving treatment, even temporary embolization can lead to uterine ischemia, increasing the risk of infection, uterine rupture in the next pregnancy [9], and Asherman’s syndrome [10]. The gelatin sponge used for UAE is absorbed in approximately two to six weeks [11], although its subsequent effects and safety during pregnancy have not been clarified.

Temporary laparoscopic clipping of the uterine artery during myomectomy effectively reduces intraoperative bleeding [12]. Imai et al. applied this technique to nine cases of cervical pregnancy by performing laparoscopic uterine artery clipping followed by hysteroscopic resection. The mean (range) surgical time, uterine artery occlusion time, and blood loss were 82 min (62-120 min), 42 min (21-68 min), and 57 ml (10-200 ml), respectively. They reported that all nine patients resumed menstruation at an average of 46 days (range, 35-80 days) after surgery, and four of the nine achieved subsequent pregnancy and delivery [13]. Temporary intraoperative occlusion of the uterine artery reduces the risk of complications due to uterine ischemia, such as infection and Asherman’s syndrome. This method potentially offers better fertility preservation than UAE.

Although temporary ligation of the uterine artery with a thread laparoscopically for retained products of conception (RPOC) [14] has also been reported, the ligation can be loosened, and releasing the ligated suture is complicated. The clip can be easily removed by using dedicated removal forceps. Therefore, clipping is easier and more likely to occlude the uterine artery.

There are few reports on hysteroscopic resection of placental polyps with laparoscopic uterine artery clipping, and our case demonstrates its efficacy. Laparoscopic uterine artery clipping does not require considerable time and is comparable to UAE in cases of severe bleeding requiring emergency intervention, allowing for a smooth transition to hysteroscopic surgery. As the incidence of placental polyps is expected to increase with an increase in ART pregnancy rates, this technique may become a favorable option for fertility preservation.

However, a limitation of this study is the lack of intraoperative assessment of decreased blood flow after clipping. It is difficult to guess how much blood would have been lost if the clipping had not been done. This procedure can be selectively applied to patients at higher risk of bleeding by assessing blood flow. In addition, the occlusion time of the uterine artery and the long-term prognosis should be followed.

## Conclusions

In conclusion, even early miscarriage requires attention to massive bleeding and the subsequent formation of placental polyps. TCR with laparoscopic temporary uterine artery clipping is an effective fertility-preserving procedure for placental polyps by avoiding long-term ischemic complications. Further studies are needed to evaluate the duration of uterine artery occlusion and post-clipping pregnancy outcomes.
